# Development of Biocompatible Bulk MgZnCa Metallic Glass with Very High Corrosion Resistance in Simulated Body Fluid

**DOI:** 10.3390/ma15248989

**Published:** 2022-12-16

**Authors:** Shi Jie Bryan Bin, Kai Soon Fong, Beng Wah Chua, Manoj Gupta

**Affiliations:** 1 Department of Mechanical Engineering, National University of Singapore, Singapore 117576, Singapore; 2 Singapore Institute of Manufacturing Technology, 73 Nanyang Drive, Singapore 637662, Singapore

**Keywords:** magnesium, amorphous, spark plasma sintering, melt spinning, mechanical properties, bio-corrosion properties

## Abstract

Magnesium-zinc-calcium (Mg-Zn-Ca) alloys as a biomaterial have attracted much attention recently, owing to their excellent biocompatibility, similar mechanical properties to natural bone, and biodegradable properties. Despite the numerous advantages of MgZnCa alloys, the rapid degradation of magnesium proved challenging as the implant in unable to retain its structural integrity for a sufficient duration of time. For metallic glasses, the capability to produce a bulk sample that is sufficiently large for useful applications have been far less successful owing to challenging processing parameters that are required for rapid cooling. In this study, Mg_65_Zn_30_Ca_5_ melt-spun ribbons were produced using melt-spinning followed by spark plasma sintering under high pressure (60 MPa) at different temperatures (130–170 °C) to provide an insight into the consolidation, mechanical, and corrosion behavior. Microstructural interfaces were characterized using scanning electron microscopy while the thermal stability of the amorphous phase was characterized using differential scanning calorimetry and X-ray diffraction. Here, pellets with 10 mm diameter and 10 mm height with a complete amorphous structure were achieved at a sintering temperature of 150 °C with densification as high at ~98%. Sintering at higher temperatures, while achieving higher densification, resulted in the presence of nano-crystallites. The mechanical properties were characterized using microhardness and compression tests. The hardness values of the sintered products were relatively higher to those containing crystallite phases while the ultimate compressive strength increased with increasing sintering temperature. Bio-corrosion properties were characterized via electrochemical testing with PBS as the electrolyte at 37 °C. The corrosion results suggest that the sintered samples have a significantly improved corrosion resistance as compared to as-cast samples. More notably, SPS150 (samples sintered at 150 °C) exhibited the best corrosion resistance (35× compared to as-cast in the context of corrosion current density), owing to its single-phase amorphous nature. This study clearly shows the potential of spark plasma sintering in consolidating amorphous ribbons to near-full density bulk pellets with high corrosion resistance for bio-applications.

## 1. Introduction

In recent years, numerous studies have been reported on magnesium-based bulk metallic glasses (Mg-BMGs) as an alternative class of material for a wide range of engineering and biomedical applications [1,2]. Although magnesium is a promising candidate for biomedical applications such as medical implants, one major limitation that is associated with magnesium is its rapid degradation in vivo. The rapid corrosion causes the Mg-based implants to degrade and lose its structural properties before the bone has healed. In addition, hydrogen gas, a by-product from the degradation of magnesium, can be entrapped due to the rapid degradation which results in localized alkalinization beneath the skin and causes tissue necrosis [3]. Owing to its amorphous structure, Mg-BMGs are of great interest in the biomedical sector as they offer a combination of enhanced properties such as biocompatibility, corrosion resistance, yield strength, and toughness [4].

Despite the advantages that Mg-BMGs hold over their crystalline counterparts, the synthesis of Mg-BMGs, or any BMGs for that matter, is an extremely challenging and demanding task as the suppression of crystalline phase requires very high cooling rates [5]. Traditional processing methods such as copper mold casting and high-pressure die casting are the most commonly used in the fabrication of Mg-BMGs. While they are quite successful in the synthesis of BMGs, they are often limited by the small dimensions of the end products, restricting any applications [5]. For instance, a recent paper by Shamlaye et al. highlighted this issue. In their work, Mg-Ag-Y-Cu BMGs were formed by copper mold casting. While they successfully showed the fabrication of BMG in their alloy composition, their casting thickness was between 1~4 mm [6]. In another study, Mg-Zn-Ca BMGs were successfully fabricated into amorphous rods with a critical diameter of 4 mm [7].

In recent years, a lot of work has been reported to enhance the glass-forming ability of Mg-based BMGs, particularly on Mg-Cu-X-X_1_ alloy systems [8,9,10]. For instance, Ma et al. successfully synthesized Mg-based BMG rods with diameters up to 25 mm with Mg-Cu-Ag-Gd using a traditional copper mold casting technique [11]. However, this process is time-consuming and environmentally unfriendly due to the large amount of material wastage just to form a small part. Table 1 summarizes some of the fabrication techniques for the development of Mg-based bulk metallic glasses (BMGs) for biomedical applications.

In the present study, a two-step synthesis approach was introduced as an alternative bulk forming process of Mg-BMGs as well as to improve on the dimensions of current Mg-BMGs by forming a larger bulk piece. This forming approach consisted of melt-spinning, followed by spark plasma sintering (SPS) at a controlled, elevated temperature. Differential scanning calorimetry (DSC) and X-ray diffraction (XRD) analysis were carried out post-processing to confirm the amorphous nature of the alloy. Mechanical properties such as micro-hardness and compressive properties were analyzed while corrosion properties were performed via electrochemical means.

## 2. Materials and Methods

### 2.1. Materials Processing

Alloy ingots with compositions of Mg_65_Zn_30_Ca_5_ were prepared by melting magnesium (Mg) turnings of 99.9% purity (ACROS Organics, Waltham, MA, USA), zinc (Zn) granules of 99.8% purity (Alfa Aesar, Haverhill, MA, USA) and calcium (Ca) granules of 99.8% purity (Alfa Aesar, USA) under protective argon gas. Disintegrated melt deposition (DMD) was the primary processing technique for the development of the master alloy.

Amorphous ribbons of the alloy were then fabricated using melt-spinning. A total of 5 g of the master alloy was processed using a crucible of quartz by melt spinning under an argon atmosphere in a single roller copper-spinning wheel. The speed of the wheel was 2000 rpm. A total of 45 MPa was set as the injection pressure while a 0.5 mm distance was used between the vial and the wheel. To increase the surface area for subsequent processing via SPS, the amorphous ribbons were then cold-compacted at room temperature with a compaction die. Using a hydraulic press, the ribbons were compacted in a 10 mm diameter die for 1 min under a pressure of 3.5 MPa.

Subsequently, the compacted ribbons were sintered into 10 mm diameter by 5 mm height disks with a high-strength carbon die via SPS at a pressure of 60 MPa at an elevated temperature between 130 and 170 °C. The SPS apparatus that was used in our work is a DR. SINTER LAB Jr. SERIES 632LX from Fuji Electronics, with a maximum sintering DC pulse of 3000 A under vacuum conditions (<3 Pa). The temperature was monitored and controlled using K-type thermocouples. The heating rate was maintained at 15 K/min before tapering it down to 7 K/min at the final stages of heating to prevent the temperature from overshooting. Table 2 shows the detailed processing parameters that were used throughout the experiment. Here, the specimens are labelled according to their processing parameters. For instance, SPS150 represents a sample that has undergone SPS at a sintering temperature of 150 °C. Once the sample has been successfully sintered, the sample is left to cool in vacuum before switching off the machine.

### 2.2. Characterization

#### 2.2.1. Surface Morphology

The surface morphology and interfaces between the melt-spun ribbons were characterized using an OLYMPUS (Shinjuku City, Japan) metallographic optical microscope as well as JEOL (Akishima, Japan) JSM-6010 scanning electron microscope (SEM). X-ray diffraction (XRD) studies with Cu K_α_ radiation and a scan speed of 2°/min were performed using the Shimadzu LAB-XRD 6000 (Kyoto, Japan) automated spectrometer for phase identification.

#### 2.2.2. Thermal Properties

Differential scanning calorimetry (DSC) analysis was performed using a Shimadzu DSC-60 instrument at a heating rate of 5 °C/min and a temperature range of 30–400 °C, under constant argon gas flow of 25 mL/min. DSC was used to identify thermal stability properties, such as the glass transition temperature (T_g_) and crystallization temperature (T_x_) which are critical parameters that need to be obtained for the SPS process.

#### 2.2.3. Mechanical Properties

A Shimadzu-HMW automatic digital microhardness tester was used to conduct microhardness measurements. Following ASTM standard E384-08, a load of 245.2 mN with 15 s dwell time was used for each indentation on the cross-sections of the SPS samples. A minimum of 20 readings were taken for each sample.

Quasi-static compression testing was conducted using an MTS 810 fully automated servo hydraulic mechanical testing machine at a strain rate of $8.33\times 10^{-5}$ s^−1^. A total of three samples with a height and diameter of 10 mm (length-to-diameter ratio of 1), as per ASTM standard E9-09 were tested.

#### 2.2.4. Bio-Corrosion Tests

The standard three-electrode set up as a potentiostat was used to conduct the electrochemical tests in phosphate buffered solution (PBS) with a pH of ~7.4 (Life Technologies, Singapore). A high-density graphite electrode was used as a counter electrode while a saturated calomel electrode was used as the reference electrode and a metal specimen was used as the working electrode. The specimen was covered with an electrochemical mask, leaving an exposure area of 1 cm^2^.

The open circuit potential (OCP) was measured for one hour. A scan rate of 1 mV/s at a voltage of ±200 mV was used for potentiodynamic polarization. The test cell was placed within a Faraday cage to minimize external electromagnetic interference. All electrochemical tests were conducted at (37 ± 0.2 °C) to simulate body conditions and repeated thrice for each specimen. The results from the electrochemical experiments were analyzed using Solartron Analytical SI 1287 Electrochemical Interface, coupled with CorrWare software version 6.33. 

## 3. Results

### 3.1. Spark Plasma Sintered Mg-Zn-Ca Bulk Metallic Glasses (BMGs)

**Figure 1:**
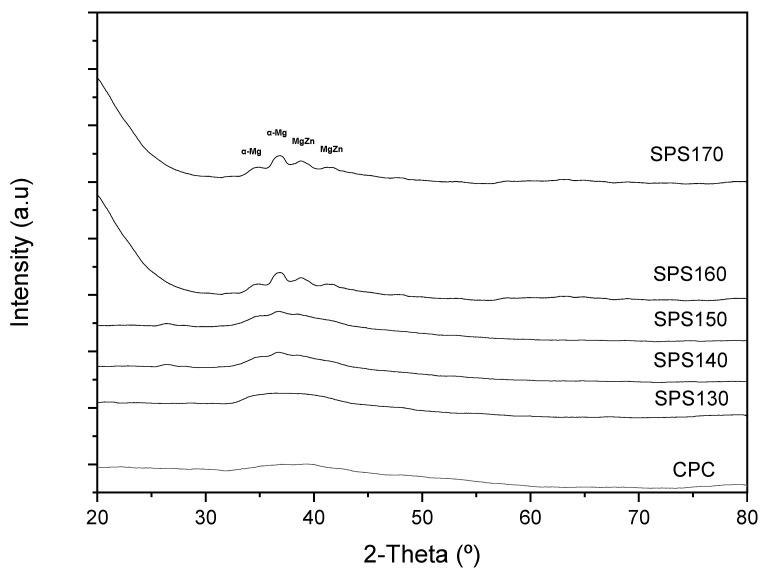
XRD patterns of pre-SPS–Cold compacted (CPC) and SPS-ed Mg-Zn-Ca ribbons at varying temperatures from 120 °C to 170 °C.

Mg_65_Zn_30_Ca_5_ BMGs were successfully sintered by SPS at various temperatures. The effect of sintering temperatures on the amorphous nature of Mg-Zn-Ca BMG can be shown by the XRD pattern in Figure 1. Up to SPS150, all the XRD curves have shown a diffused broad peak at ~2θ = 38°, indicating the amorphous nature of the specimen. Above 150 °C, minor peaks, corresponding to Mg-Zn and α-Mg crystal phases were observed, indicating that some crystallization has occurred. The results also show that in SPS140 and SPS150, the intensity of the broad peaks increased relative to the spectra, forming very minute peaks at 2θ = 37°, across the diffused regions. This behavior can be attributed to some form of structural relaxation that occurred when heating up to a temperature above the glass transition temperature, resulting in a more orderly atomic distribution while limiting the ability for long-range diffusion of atoms that happens more evidently at the crystallization temperature. 

**Figure 2:**
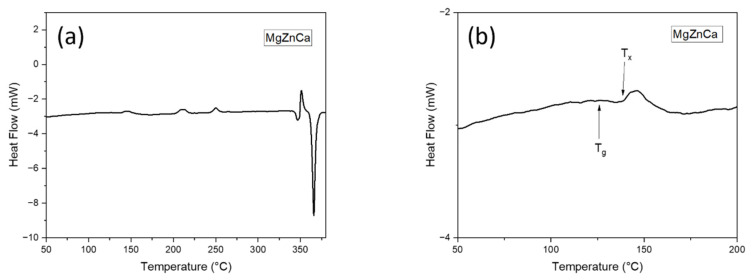
DSC curves of the SPS-ed Mg-Zn-Ca Melt Spun ribbons: (**a**) 50–400 °C and (**b**) 50–200 °C showing both the glass transition temperature (T_g_) and crystallization temperature (T_x_).

This phenomenon can further be supported by the DSC results. Figure 2a shows the DSC trace pattern corresponding to the melt spun Mg_65_Zn_30_Ca_5_ ribbons, at a heating rate of 5 °C/min. With increasing temperature, Figure 2b shows glass transition, supercooled liquid region, followed by crystallization, as evident by an exothermic peak. The glass transition temperature, T_g_, is the temperature at which an amorphous solid undergoes a second-order transition where the atoms begin to achieve sufficient thermal energy for short-range diffusion [15] while crystallization temperature T_x_ is the temperature at which the atoms are able to gain sufficient energy for long-range diffusion, thereby re-arranging themselves into an orderly manner, and start to crystallize [16].

From Figure 2b, it can be observed that the T_g_ and T_x_ for the melt spun Mg_65_Zn_30_Ca_5_ ribbons is ~130 °C and ~150 °C, respectively. This further validates the accuracy of the XRD results and possibly be the reason for the minute changes in the board diffused peak between SPS130 and SPS140. At sintering temperatures of 140 °C and 150 °C (SPS140 and SPS150), it is well beyond the glass transition temperature of 130 °C. As such, the atoms are able to achieve sufficient energy for short-order atomic arrangement, resulting in some form of structural relaxation and disrupt the broad diffused peaks, yet maintain an amorphous nature.

**Figure 3:**
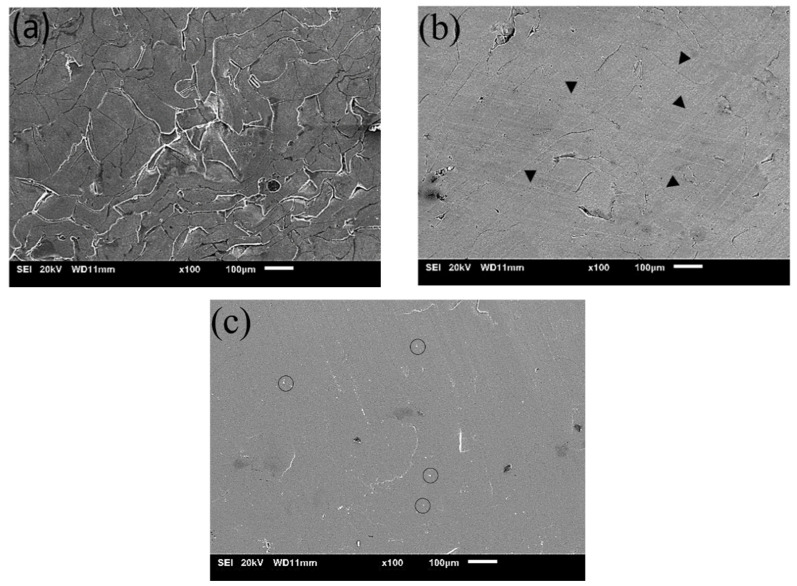
SEM micrographs of (**a**) pre-SPS Mg-Zn-Ca BMG and SPS Mg-Zn-Ca BMG at (**b**) 150 °C and (**c**) 160 °C with melt spun ribbons at 60 MPa pressure.

The surface morphology corresponding to pre-SPS–Cold compacted (CPC), SPS150, and SPS160 is shown in Figure 3. Comparing Figure 3a,b it is evident that SPS as a post-processing technique is successful in creating a bulk sample from the melt-spun ribbons. The interfaces between the ribbons which were obvious pre-SPS were significantly reduced, suggesting that interfacial bonding had taken place during SPS.

Denoted by arrows in Figure 3b, the geometrical features of possible ribbon interfaces that are no longer present are shown. This could indicate some form of mechanical interlocking between the ribbons. In addition, a relatively small volume fraction of pores as compared to CPC (1.98 vol.%) could be observed from the micrograph while no obvious contrast features that can be attributed to the presence of crystallinity can be seen.

Figure 3c represents the surface morphology for SPS160, which is above the crystallization temperature of 150 °C. Comparing Figure 3b,c, the ribbon interfaces for SPS160 can be observed to be further reduced, resulting in a smooth and homogeneous surface with little porosity (1.72 vol.%) and some second phase particles as suggested by the circles. This can be attributed to a higher sintering temperature, particularly above the crystallization temperature where atoms are able to undergo long-range and quicker diffusion, thereby increasing interfacial bonding, decreasing porosity, and resulting in the formation of fine precipitates.

### 3.2. Density and Porosity

**Figure 4:**
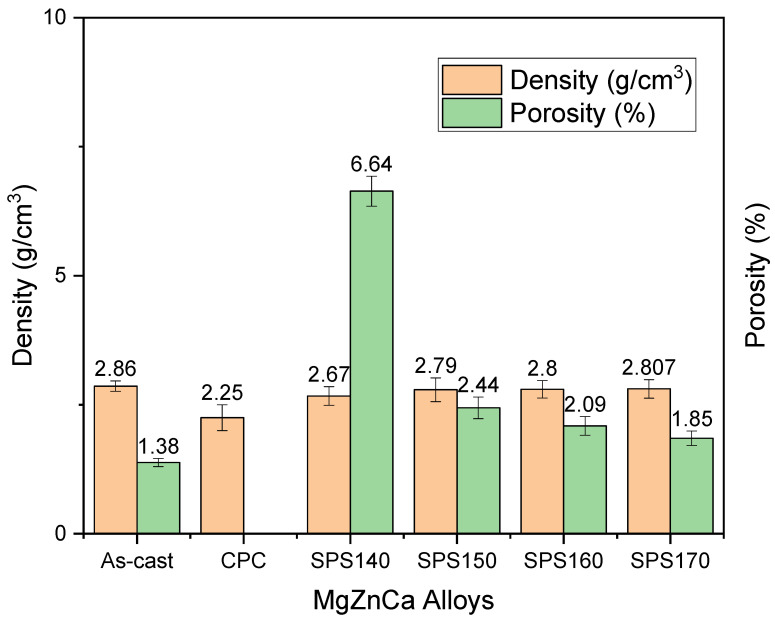
Density and porosity of Mg-Zn-Ca BMGs post-processing as compared to as-cast.

The measured density and porosity of Mg-Zn-Ca BMGs compared to as-cast is shown in Figure 4 below. The experimental densities were calculated using Archimedes’ principle. A total of three samples were used to measure the densities of the materials using an A&D GH-252 electronic. Here, the results have shown the near net shape capability of SPS as a processing technique while maintaining the amorphous structure. The densities of SPS140, SPS150, SPS160, and SPS170 were 2.67, 2.79, 2.80, and 2.807 g/cm^3^, respectively, while their porosities were 6.64, 2.44, 2.09, and 1.85 %, respectively. When compared to the as-cast Mg-Zn-Ca master alloy that has a density of 2.86 g/cm^3^ and porosity of 1.38%, SPS have shown to be successful in sintering the melt-spun ribbons into BMGs while maintaining the amorphous nature as shown in SPS150. Further, the porosities of SPS-ed samples, particularly at 150–170 °C, remained nearly in the range of near net shape products (~2%).

### 3.3. Mechanical Properties

**Figure 5:**
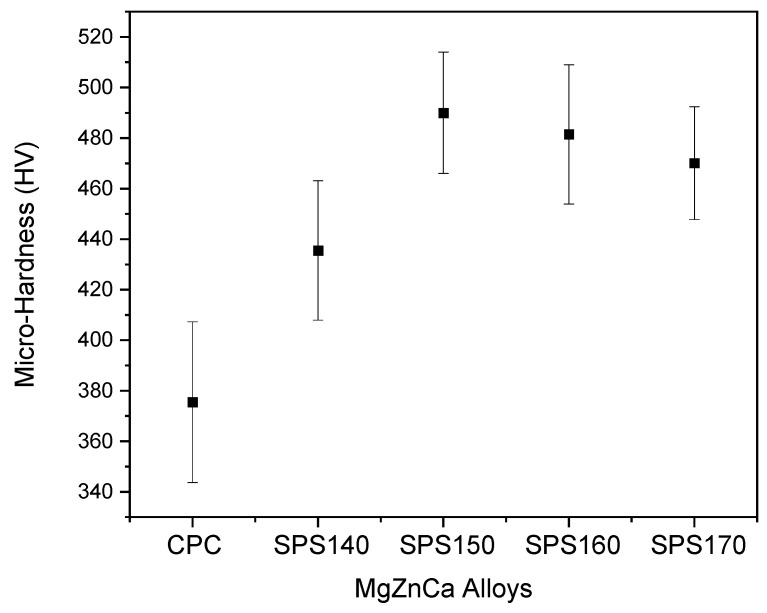
Micro-hardness of Mg-Zn-Ca BMGs.

The mechanical response in this study was assessed from micro-hardness in Figure 5. Micro-hardness increased with increasing sintering temperature and tapered off after SPS150, with a maximum hardness value of 491 HV exhibited by SPS150 samples. This increase in micro-hardness from CPC to SPS150 can be attributed to an increase in the sample density and a reduction in the porosity. From SPS150 to SPS170, micro-hardness starts to taper off. From SPS150 to SPS160, the material changes from a fully amorphous structure to one with growth crystalline precipitates as shown in the XRD pattern in Figure 1. As such, the ductility may have increased because of crystalline growth, leading to a drop in hardness value [10].

**Figure 6:**
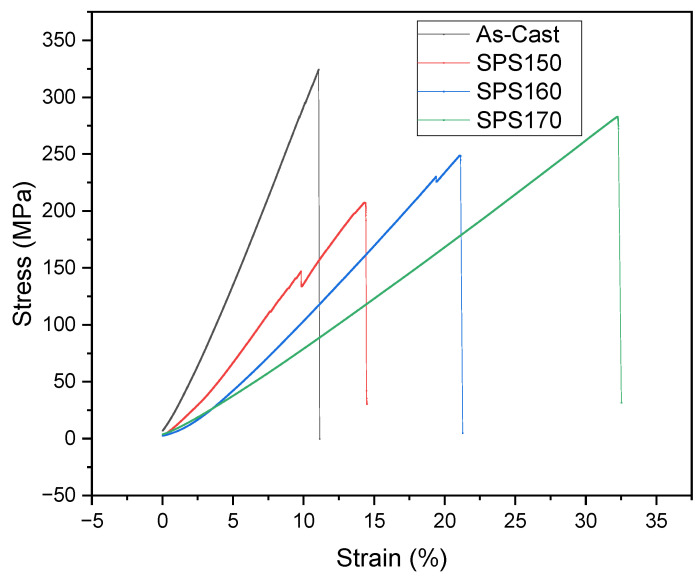
Compressive stress-strain curves of Mg-Zn-Ca BMGs compared to as-cast.

The mechanical response of Mg-Zn-Ca BMGs was also studied and compared with as-cast samples via room temperature compressive testing. The uniaxial compressive engineering stress-strain curves are shown in Figure 6 and the results are presented in Table 3. The results revealed that both the yield and compressive strength of the as-cast Mg-Zn-Ca were higher than that of the SPS-ed samples. This may be due to the presence of porosity in the SPS-ed samples which can act as localized points of crack initiation [17], resulting in the premature failure of a material. More studies will be carried out to reduce porosity, thereby improving densification and mechanical properties.

Between the SPS-ed samples, an improvement in the ultimate compressive strength (UCS) and fracture strain can be seen with increasing sintering temperature, with a maximum UCS of 288 MPa exhibited by SPS170 samples with significant ductility at 32%. Notably, the mechanical properties of SPS-ed samples are superior to that of cortical bone, with a compressive strength of 205 MPa [18]. Of note is the similarity in compressive strength can negate the effect of stress shielding [19] which often occurs due to a mismatch in mechanical properties between the implant material and bone [20].

### 3.4. Bio-Corrosion Properties

**Figure 7:**
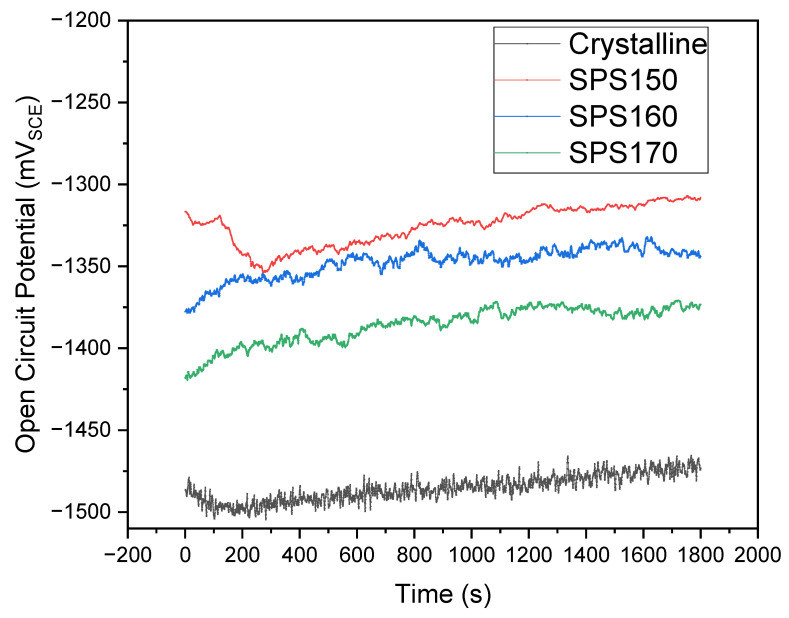
Time dependence open circuit potential (OCP) across various sintering temperatures compared to as-cast.

**Figure 8:**
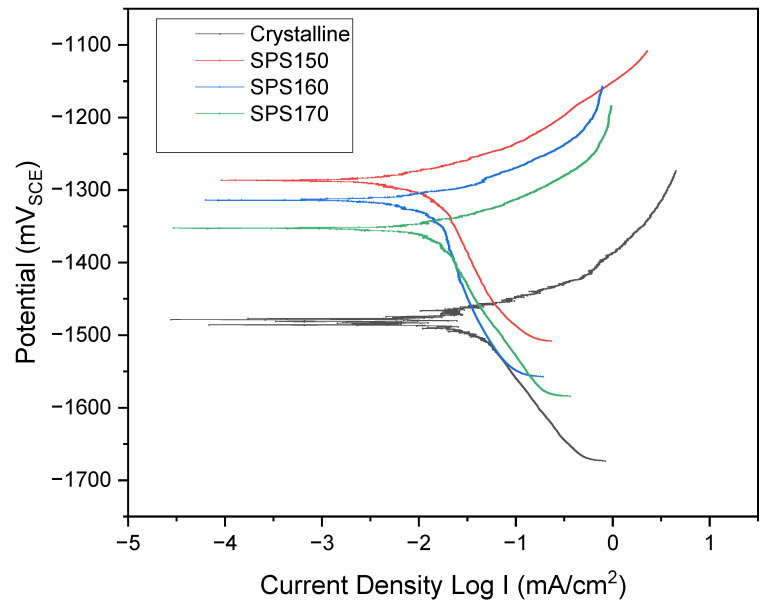
Potentiodynamic plot of Mg-Zn-Ca across various sintering temperatures compared to as-cast.

The time-dependence of open circuit potential (OCP) and potentiodynamic polarization curves of the samples immersed in PBS solution at 310 K are shown in Figure 7 and Figure 8, respectively. In the initial stages of OCP, the samples shift towards a more positive potential and stabilizes, suggesting the formation of a passive film on the surface. The potential during OCP is relatively stable for the SPS-ed samples while the crystalline sample exhibits significant fluctuation in potential across the entire duration of the OCP. This would suggest some form on instability such as de-passivation and re-passivation of the surface film and the occurrence of pitting corrosion that may have occurred during the OCP [21].

In Figure 8, the polarization curves for the SPS-ed samples and crystalline show a similar profile and exhibit current plateaus that are associated with the formation of passive film at the anodic region. However, the crystalline Mg-Zn-Ca shows significantly quicker dissolution at the anodic region, depicted by the steeper anodic gradient and higher corrosion current densities at larger potentials.

At a potential of ~−1500 mV, the crystalline Mg-Zn-Ca exhibits a ‘zig-zag’ polarization section which can be an indication of non-uniform corrosion such as pitting corrosion while the SPS-ed bulk metallic glasses generally exhibit more uniform corrosion.

The rate of corrosion can be related to potential too, i.e., a material having more positive potential acts as a noble material and shows higher corrosion resistance. Table 4 shows the average corrosion current density (I_corr_) and potential of the specimens. The corrosion current density is a direct relation to the corrosion rate, that is, a larger corrosion current density would suggest a higher dissolution of metal, resulting in a higher corrosion rate [22]. Here, the data suggest that bulk metallic glasses have significantly better corrosion resistance as compared to its crystalline counterpart. When comparing crystalline Mg-Zn-Ca to SPS150, I_corr_ values suggest ~35× lower current density in SPS150 (321.5 × 10^−4^ mA/cm^2^ vs. 9.14 × 10^−4^ mA/cm^2^).

It is interesting to note that with increasing sintering temperatures (SPS160 and SPS170), corrosion current density increases to 18.3 × 10^−4^ mA/cm^2^ and 24.3 × 10^−4^ mA/cm^2^, respectively. The increase in corrosion current density can be attributed to the formation of crystalline phases in SPS160 and SPS170 that were not present in SPS150, as suggested by the XRD plot in Figure 1.

The development of precipitate phases directly affects corrosion rate in Mg-based alloys. The phases that were identified in Figure 1 consist of Mg-Zn which is generally more noble than pure Mg [23], resulting in the formation of mini galvanic cells on the surface of the material and increasing the corrosion rate significantly. Similar results were also seen in other Mg-multicomponent alloys. For instance, Zhou et al. observed MgAlCu phases that act as micro-cathodic sites and was the primary cause of rapid degradation in AZ31-Cu alloys [24]. In a separate paper by Song and Xu, the presence of Al-Mn intermetallic phases in AZ31 Mg alloy have shown to lead to rapid dissolution [25]. Therefore, when the volume fraction of precipitates increases, the corrosion rate would increase as well.

This agrees with our studies where SPS160 has a lower corrosion rate than SPS170. At higher sintering temperatures, the volume fraction of precipitates would increase due to the increase in the rate of diffusion and mass-transfer. As such, the corrosion activity will increase due to the formation of more galvanic cells per unit area. SPS150 on the other hand, exists as a single-phase amorphous alloy with no crystalline phases detected. Therefore, galvanic corrosion should be very limited and has the least corrosion rate among the sintered samples.

## 4. Discussion

### Influence of Spark Plasma Sintering as a Post-Processing Technique on Melt-Spun Ribbons

The issue with any sintering technique used is the ability to form good interfacial bonding between powder particles/ribbons. This is particularly tricky for amorphous metals as a good glass-forming system should limit mass-transfer, thereby hindering diffusivity [26]. To improve diffusion, there are a few parameters such as the temperature, pressure, and surface area of particles that can be controlled. While increasing the temperature increases the rate of mass-transfer, thereby improving compaction and forming better interfacial bond, too high a temperature will result in crystallization and destroy the amorphous nature as shown in SPS160 and SPS170 in our experiment. Therefore, there is a limit to how high the temperature can be raised during the sintering process.

To overcome the low diffusion rate in an amorphous matrix, we can increase the sintering pressure, forcing atoms to diffuse across barriers. For instance, increasing pressure can mechanically shear and induce brittle fracture in metallic glass ribbons/particles that have very little ductility to begin with [27]. This would result in an increased surface area and density for surface contact during the sintering process.

The utilization of high pressure can thus be used to overcome the limitations that are set forth by temperature, which is controlled by T_x_. High pressure densification during SPS has been studied in detail. For instance, the work that was done by Zheng et al. on Mg-Cu-Gd alloy has shown that the densification rate, $\frac{dv_{d}}{dt}$, can be governed by the following equation [28]:

$$\frac{dv_{d}}{dt}=\left(1-v_{s}\right)B\left(g\frac{\gamma_{sv}}{x}+P_{e}\right)$$

where $\left(1-v_{s}\right)$ represents the fractional porosity, B is a material dependent diffusion parameter, $\gamma_{sv}$ is the solid-vapor interfacial energy, *g* and *x* are geometrical terms that depends on the final product’s geometry such as porosity, while *P_e_* is the pressure that is exerted during the sintering step. With increasing applied pressure, the rate of densification increases due to the acceleration of viscous flow and tapers off as near-full density is achieved.

In the present study, 60 MPa is the maximum pressure that could be exerted due to constraints that are brought forth by the equipment as well as the compaction die that was used. Notably, there have been reports of 400–500 MPa being utilized with industrial grade SPS equipment, such as in a paper that was published by Perriere et al. with the utilization of 500 MPa, where full densification of amorphous powders was realized [29]. Since melt-spun ribbons have lesser surface area than powder, it would be interesting to see if full densification can be obtained with melt-spun ribbons at higher pressures, given that gas atomization of powders generally tends to be much more expensive, time consuming, and complex [30].

Increasing the surface area for interfacial diffusion can also be used to improve densification. For gas atomized Zr-based amorphous powder, Perriere et al. managed to achieve near 100% densification when sintered at its glass transition temperature [29]. Similar reports were published in Mg-based amorphous power by Sabina et al. [31] and Zheng et al. [28] as well as Ni-based amorphous powder by Ye et al. [32]. However, despite sintering at a temperature that is slightly below the crystallization temperature of their respective alloy systems, partial devitrification and precipitation occurred in all their SPS-ed powder samples. While in the case of our SPS-ed melt spun ribbons, no precipitation has occurred when sintering slightly below the crystallization temperature as seen in SPS150.

**Figure 9:**
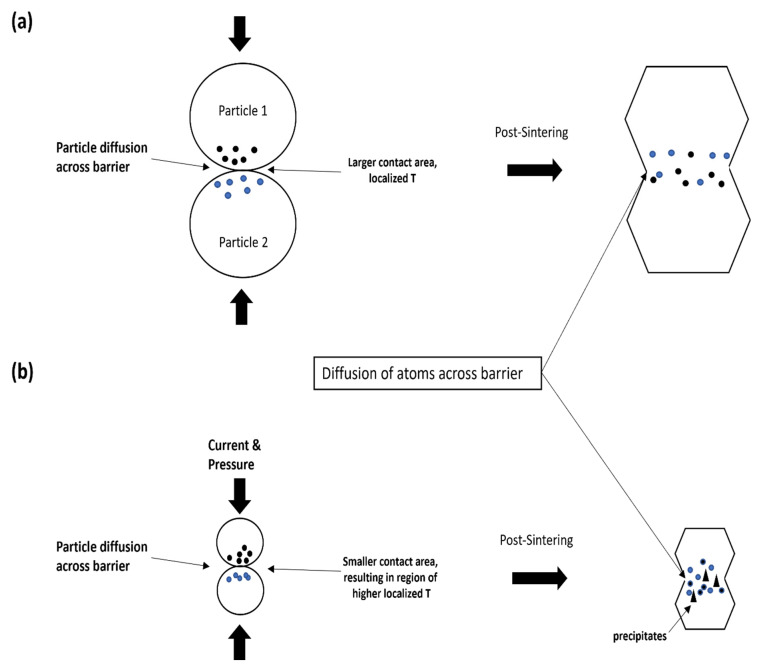
Schematic illustration of the compaction mechanism via SPS of (**a**) larger particles as compared to (**b**) finer particles.

This may be due to size of the particles during the sintering process. Due to the significantly smaller size of powder, the contact between two surfaces during SPS is reduced significantly, as shown in Figure 9b. As such, localized heat that is generated at the initial particle necks is extremely high as compared to the initial sintering temperature, resulting in high thermal diffusivity (better compaction, ~99–100%) but partial devitrification and crystallization at the necks where the temperature is higher than the crystallization temperature.

As a result, semi-amorphous structures are usually formed. SPS-ed melt spun ribbons on the other hand, have a larger surface area of contact between ribbons during the sintering process, where the increase in localized temperature at the necks is potentially not as high in the amorphous powder. This could explain why we are able to maintain the full amorphous structure post-sintering, despite having a sintering temperature just below the crystallization temperature while having a reasonable amount of densification (~98 % densification in SPS150 as compared to its master alloy).

## 5. Summary

The influence of melt-spinning and SPS on the fabrication of Mg-Zn-Ca bulk metallic glass was studied. The microstructure, interfacial bonding, mechanical, and bio-corrosion response of SPS-ed specimens were investigated. The experimental results revealed the following:

(1) SPS can be effectively used as a sintering technique to produce Mg-Zn-Ca bulk metallic glass at elevated temperature (130–170 °C) and high pressure (60 MPa).
(2) A fully amorphous structure was obtained post-sintering at a temperature near its crystallization temperature as shown in SPS150, with good amount of densification compared to the master alloy (2.79 g/cm^3^ vs. 2.86 g/cm^3^, ~98% densification). Densification increases with increasing sintering temperature.
(3) SPS-ed samples achieved a maximum ultimate compressive strength of 288 MPa as compared to as-cast with an ultimate compressive strength of 324 MPa. The mechanical properties of SPS-ed samples increased with increasing sintering temperature and remained higher than that of cortical bone.
(4) SPS-ed samples were shown to have significantly improved corrosion resistance in SBF with the smallest corrosion current density of 9.14 × 10^−4^ mA/cm^2^ with SPS150 compared to the master alloy with 321.5 mA/cm^2^. The presence of crystallinity in SPS160 and SPS170 increased the corrosion rate but it remained significantly lower than the corrosion rate of the master alloy.

In summary, the bulk consolidation of melt-spun ribbons opens interesting perspectives to produce Mg-based bulk metallic glasses for a wide range of biomedical applications. On one hand, a higher sintering temperature would result in better mechanical properties due to greater densification but a higher corrosion rate due to the presence of precipitates that are formed. While on the other, sintering at a lower temperature such as near its crystallization temperature (SPS150) results in a complete amorphous matrix thereby improving the corrosion resistance but resulting in relatively poorer mechanical properties due to lesser densification. As such, the sintering parameters can be adjusted accordingly to suit the needs of the application.

## Figures and Tables

**Table 1:** Summary of reported synthesis routes of Mg-based BMGs.

Name of Mg-Based Bulk Metallic Glass (Composition)	Production/Synthesis Method	Post-Production Structure	Diameter (mm)	References
Mg_65_Cu_20_Y_10_Zn_5_ Mg_75_Cu_13.3_Y_6.6_Zn_5_	Inert atmosphere /Induction furnace	Fully homogeneous amorphous and heterogenous structure	3	[12]
Mg_60_Zn_35_Ca_5_	Process of powder metallurgy	All amorphous phase	10	[13]
Mg_66_Zn_30_Ca_4_	Process of powder metallurgy followed by Cy casting	All amorphous phase	2–10	[14]
Mg_58.5_Cu_30.5_Y_11_	Arc melting followed by direct casting	All amorphous phase	16–25	[11]

**Table 2:** Detailed experimental procedure used in spark plasma sintering.

Specimen No.	Starting Temperature (°C)	Heating Rate (°C/min)	Final Temperature (°C)	Heating Rate (°C/min)	Sintering Temperature (°C)	Dwelling Time (min)	Pressure (MPa)
SPS130	30	15	90	7	130	15	60
SPS140	30	15	90	7	140	15	60
SPS150	30	15	90	7	150	15	60
SPS160	30	15	90	7	160	15	60
SPS170	30	15	90	7	170	15	60

**Table 3:** Results of compressive testing of Mg-Zn-Ca BMGs compared to as-cast and cortical bone.

Sample Name	UCS (MPA)	Fracture Strain (%)	Energy Absorbed (mJ/mm^3^)	Reference
as-cast	324 ± 10	11.1 ± 0.98	17.3 ± 2.6	-
cortical bone	205 ± 17	1.3 ± 0.3		[18]
SPS150	207 ± 11	14.3± 0.9	14.8 ± 1.8	-
SPS160	248 ± 10	21.1 ± 1.6	24.4 ± 2.6	-
SPS170	288 ± 16	32.2 ± 4.0	44.3 ± 4.9	-

**Table 4:** Comparison of I_corr_ and E_corr_ between Mg-Zn-Ca across various sintering temperature compared to as-cast.

Sample Name	I_corr_ (mA/cm^2^)	Improvement in Corrosion Resistance Based on I_corr_	E_corr_ (mV_SCE_)	Corrosion Rate (mm/year)
Crystalline	321.5 × 10^−4^	-	−1467	0.688
SPS150	9.14 × 10−4	35×	−1287	0.00672
SPS160	18.3 × 10^−4^	17.5×	−1298	0.0392
SPS170	24.3 × 10−4	13×	−1351	0.0520

## Data Availability

Not applicable.
